# The Hydrophobic Patch Directs Cyclin B to Centrosomes to Promote Global CDK Phosphorylation at Mitosis

**DOI:** 10.1016/j.cub.2019.12.053

**Published:** 2020-03-09

**Authors:** Souradeep Basu, Emma L. Roberts, Andrew W. Jones, Matthew P. Swaffer, Ambrosius P. Snijders, Paul Nurse

**Affiliations:** 1 Cell Cycle Laboratory, The Francis Crick Institute, London NW1 1AT, UK; 2 Protein Analysis and Proteomics Platform, The Francis Crick Institute, London NW1 1AT, UK; 3 Laboratory of Yeast Genetics and Cell Biology, Rockefeller University, New York, NY 10065, USA

**Keywords:** SPB, centrosome, cyclin, localization, mitosis, CDK

## Abstract

The cyclin-dependent kinases (CDKs) are the major cell-cycle regulators that phosphorylate hundreds of substrates, controlling the onset of S phase and M phase [1, 2, 3]. However, the patterns of substrate phosphorylation increase are not uniform, as different substrates become phosphorylated at different times as cells proceed through the cell cycle [4, 5]. In fission yeast, the correct ordering of CDK substrate phosphorylation can be established by the activity of a single mitotic cyclin-CDK complex [6, 7]. Here, we investigate the substrate-docking region, the hydrophobic patch, on the fission yeast mitotic cyclin Cdc13 as a potential mechanism to correctly order CDK substrate phosphorylation. We show that the hydrophobic patch targets Cdc13 to the yeast centrosome equivalent, the spindle pole body (SPB), and disruption of this motif prevents both centrosomal localization of Cdc13 and the onset of mitosis but does not prevent S phase. CDK phosphorylation in mitosis is compromised for approximately half of all mitotic CDK substrates, with substrates affected generally being those that require the highest levels of CDK activity to become phosphorylated and those that are located at the SPB. Our experiments suggest that the hydrophobic patch of mitotic cyclins contributes to CDK substrate selection by directing the localization of Cdc13-CDK to centrosomes and that this localization of CDK contributes to the CDK substrate phosphorylation necessary to ensure proper entry into mitosis. Finally, we show that mutation of the hydrophobic patch prevents cyclin B1 localization to centrosomes in human cells, suggesting that this mechanism of cyclin-CDK spatial regulation may be conserved across eukaryotes.

## Highlights

• The hydrophobic patch of human and yeast cyclin B directs it to the centrosome
• Loss of the yeast cyclin B hydrophobic patch allows S phase but prevents mitosis
• Compartmentalized mitotic CDK phosphorylation relies on the hydrophobic patch

## Graphical Abstract

**Figure:**
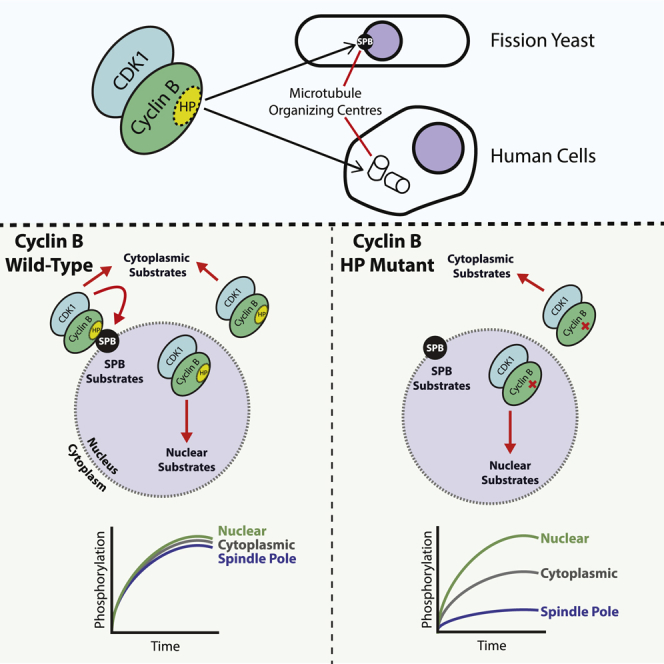

## Teaser

Cyclin-CDK accumulates on mitotic microtubule-organizing centers. Basu et al. find that the substrate-docking region of cyclin B, the hydrophobic patch, governs this localization in both human cells and fission yeast. Without the cyclin B hydrophobic patch, CDK phosphorylation is impaired at mitosis but only in certain subcellular compartments.

## Results and Discussion

All eukaryotes have multiple cyclin-dependent kinase (CDK) complexes, and it is generally thought that qualitative differences in substrate specificity between the different complexes form the basis for ordering cell cycle progression [1, 4, 5]. However, cyclin-CDK complexes exhibit considerable functional redundancy, suggesting they share extensive overlap in substrate specificities [8, 9, 10, 11]. In the fission yeast *Schizosaccharomyces pombe*, all mitotic and meiotic cyclin-CDK complexes can be replaced by a single cyclin-CDK composed of the protein kinase CDK1 (Cdc2) and the mitotic B-type cyclin Cdc13, fused as a monomer, and expressed from the Cdc13 promoter [6, 7]. This Cdc13-CDK1 fusion oscillates similarly to Cdc13 during the cell cycle, increasing in level throughout the cell cycle before being degraded at mitosis [6]. These results support the view that a progressive quantitative increase in CDK activity through the cell cycle underlies the temporal order of cell cycle events. This model is further supported by phosphoproteomic studies showing that the combination of a continuous increase of *in vivo* CDK activity through the cell cycle, together with differential substrate sensitivities to CDK activity, drives orderly cell cycle progression [5, 12]. Differences in CDK activity toward different substrates could be generated by inherent properties of the substrate, cyclin-CDK localization, or by targeting of cyclin-CDK to specific substrates [13, 14].

The localization of CDK is known to be a major determinant of how mitotic CDK complexes generate differential activity toward different substrates [14, 15]. Cyclin B1-CDK localization to the mammalian centrosome and fission yeast spindle pole body (SPB) is thought to increase local concentrations of CDK for the phosphorylation of substrates [16, 17]. Within mitosis, cyclin B1-CDK localizes at kinetochores, centrosomes, chromatin, and nuclear pores, where it is known to phosphorylate key mitotic substrates [18, 19, 20, 21, 22]. In addition, the re-localization of human cyclin B2 from the Golgi body to the cytoplasm gives cyclin B2 the ability to reorganize cytoplasmic microtubules at mitosis, a function usually restricted to the cytoplasmic cyclin B1 [23].

A further mechanism for generating differential CDK activity toward different substrates has been shown for the budding yeast *Saccharomyces cerevisiae* based on docking interactions between S-phase substrates and S-phase cyclins *in vitro* due to a conserved cyclin hydrophobic patch that interacts with R/KxL motifs in CDK substrates [1, 4, 24, 25, 26]. It was proposed that this targeting results in preferential CDK activity toward S-phase substrates, providing a potential mechanism to order cell cycle events. Mutation of the hydrophobic patch reduces the phosphorylation of S-phase substrates by S-phase cyclin-CDK complexes *in vitro*, but mutation of equivalent residues in M-phase cyclins does not influence mitotic cyclin-CDK phosphorylation of the same substrates [1]. Although substrate-docking interactions have been shown to be important for the phosphorylation of some M-phase cyclin targets, mitotic cyclin-CDK complexes possess higher intrinsic kinase activity and are thought to be less reliant on docking interactions for substrate phosphorylation [4, 27].

### The Cdc13 Hydrophobic Patch Is Not Necessary for S Phase

**Figure 1:**
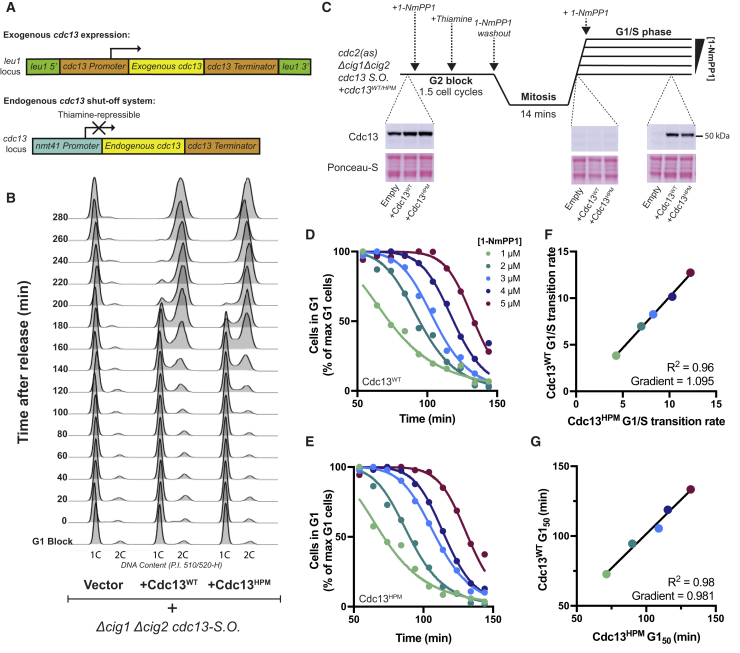
The Cdc13 Hydrophobic Patch Is Not Necessary for S Phase (A) Promoter systems used in (B)–(G) to repress endogenous *cdc13*^+^ through the thiamine-repressible *nmt41* promoter. An extra copy of *cdc13* is inserted into the *leu1* (exogenous) locus, maintaining all endogenous UTR regions. (B) Cells lacking the G1/S cyclin genes *cig1*^*+*^ and *cig2*^*+*^, with *cdc13*^*HPM/WT*^ in the *leu1* locus, were arrested in G1 through nitrogen starvation. Thiamine was added to cultures 1 h before release. Cells were then released into S phase at 32°C through refeeding minimal media containing nitrogen and examined for DNA content (see STAR Methods). S.O., shut off. (C) Upper: experimental schematic for (D)–(G). Cells are blocked in G2 initially using 1 μM of the ATP analog 1-NmPP1. Thiamine is added to repress endogenous *cdc13* 1 h before release (see STAR Methods). Cells are then washed of 1-NmPP1 to release into mitosis before addition of a range of 1-NmPP1 concentrations 14 min after the washout to study S phase by DNA content analysis. Lower: western blots show complete degradation of endogenous Cdc13. Cdc13 levels (upper) and total protein amounts (Ponceau-S, lower) before the addition of 1-NmPP1, 20 min after mitosis and 120 min after mitosis are shown from left to right, respectively. Western blots are of cells released into 3 μM 1-NmPP1. Empty corresponds to cells lacking any exogenous *cdc13* construct in the *leu1* locus. All Cdc13 and Ponceau-S panels are from the same exposure of a single membrane. (D and E) G1 cells after S phase release as a percentage of the G1 cell population at 54 min after mitosis (see STAR Methods). Before 54 min, cells are present as binucleate septated cells and not uninucleate G1 cells. (D) Cdc13^WT^ and (E) Cdc13^HPM^ are shown. Curves are a sigmoid fit through the data. (F and G) Comparison of (F) the population-level G1/S transition rate, measured as the exponent of the sigmoid fit curves in (D) and (E), and (G) the time at which 50% of cells have executed S phase as measured by G1_50_ values extracted from the sigmoid fit curves in (D) and (E). See also Figure S1 and Table S2.

Given that the hydrophobic patch is conserved among mitotic cyclins and has been shown to aid phosphorylation of S-phase substrates by S-phase cyclins, we tested whether the hydrophobic patch was necessary for ordering the cell cycle in a strain where both S phase and mitosis are driven by a mitotic cyclin. We therefore constructed a hydrophobic-patch mutant (HPM) of the mitotic cyclin Cdc13 with 3 previously characterized substitutions (M235A, L239A, and W242A) [25]. These mutations are known to disrupt substrate binding to the hydrophobic patch without reducing intrinsic CDK activity [1, 4, 25]. Cdc13^HPM^ was introduced in an exogenous locus, not fused to Cdc2, into a strain deleted for the G1/S cyclins *cig1* and *cig2* and the expression of endogenous *cdc13* placed under control of a thiamine-repressible promoter (Figure 1A) [28]. Although, in wild-type cells, the G1/S cyclins Cig1 and Cig2 are expressed in G1 to execute DNA replication, in this situation, Cdc13 is expressed in G1 and compensates for their loss [8].

To test whether Cdc13^HPM^ was able to execute DNA replication *in vivo*, cells were arrested in G1 in the presence of thiamine, repressing endogenous Cdc13, and released into S phase. Unexpectedly, cells with Cdc13^HPM^ were found to enter into and undergo S phase with similar timing to cells with Cdc13^WT^ (Figure 1B). This S phase was found to be functional, as cells went on to produce viable colonies when endogenous Cdc13 function was restored after a single round of Cdc13^HPM^-dependent S phase (Figures S1A–S1C). We also tested whether differential sensitivity between Cdc13^HPM^ and Cdc13^WT^ to the CDK inhibitor Rum1 or the inhibitory Wee1 kinase was masking potential differences between Cdc13^HPM^ and Cdc13^WT^. However, we found no difference in S phase progression between Cdc13^HPM^ and Cdc13^WT^ in the absence of Rum1 (Figure S1D) and found that Wee1-dependent CDK-Y15 phosphorylation was similar between Cdc13^HPM^-CDK and Cdc13^WT^-CDK (Figure S1E).

Although Cdc13^HPM^-CDK can execute a functional S phase, there could be a defect in S phase onset that is masked by rapidly rising CDK activity [5]. To test for subtle activity differences between Cdc13^WT^-CDK and Cdc13^HPM^-CDK, we exaggerated potential differences in CDK activity by using an analog-sensitive allele of CDK (*cdc2*^as^) [29, 30] and adding increasing doses of an inhibitor, 1-NmPP1, before S phase onset. By degrading endogenous Cdc13 completely in the mitosis prior to S phase (Figure 1C), we found that cells driven either by Cdc13^WT^ or Cdc13^HPM^ alone exhibited an identical dose-responsive S phase delay to 1-NmPP1, as measured by the time taken for 50% of cells to enter S phase (Figures 1C–1G). We conclude that a functional Cdc13 hydrophobic patch is not required for S phase, and therefore, this mechanism cannot form the basis for ensuring that S phase occurs early in a cell cycle driven solely by a mitotic cyclin. In addition, ablation of the Cdc13 hydrophobic patch likely causes no intrinsic defects in CDK activity, because S phase progresses unhindered.

### Mutation of the Hydrophobic Patch Results in Defective Cdc13 Localization

**Figure 2:**
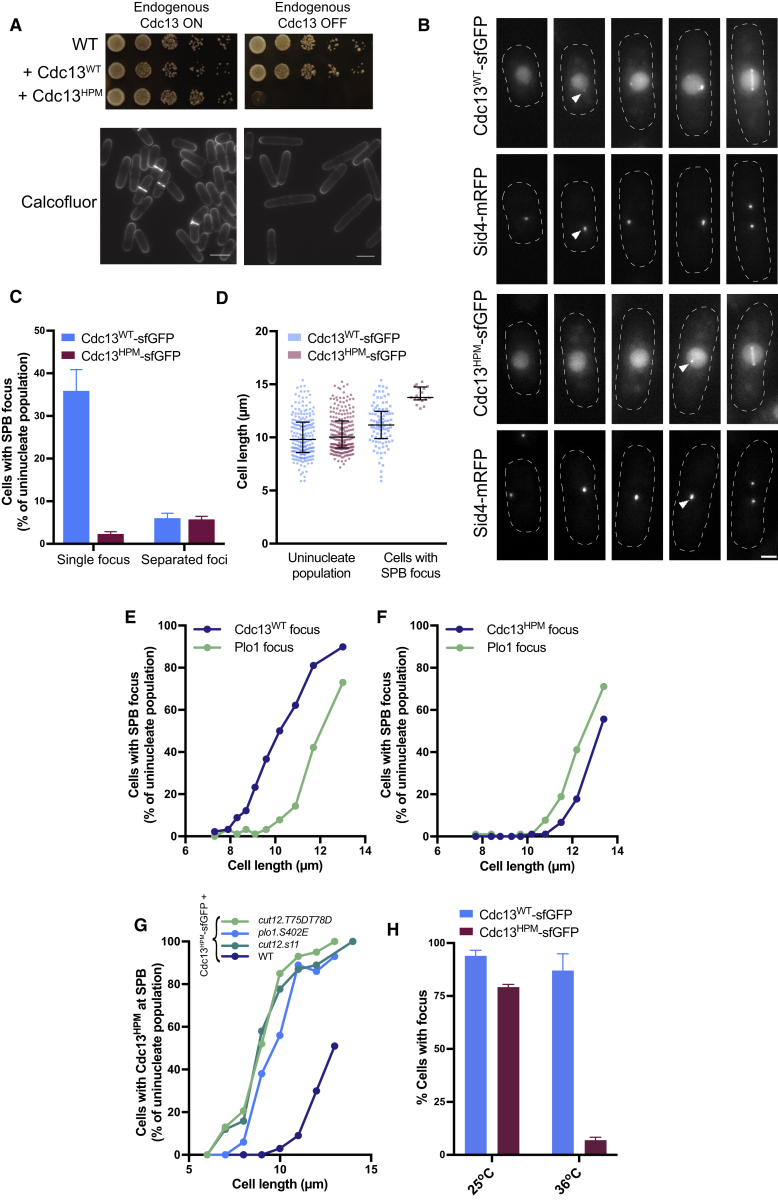
Cdc13^HPM^ Cannot Localize to the SPB in Interphase (A) Top: serial dilution assays of cells with thiamine-repressible endogenous Cdc13 and either Cdc13^WT^ or Cdc13^HPM^ in the presence (endogenous Cdc13 OFF) or absence (endogenous Cdc13 ON) of thiamine. Bottom: calcofluor staining of cells expressing Cdc13^HPM^ and repressible endogenous Cdc13 in the presence (right) or absence (left) of thiamine is shown. Scale bars, 10 μm. (B) Representative maximum projection images of different cells of increasing size from an asynchronous population expressing exogenous Cdc13^WT/HPM^-sfGFP. Endogenous Cdc13 is still expressed but is not fused to a fluorophore. Sid4-mRFP marks the SPB. Arrows show first appearance of a Cdc13-sfGFP SPB focus. The same pixel range has been applied to all images from the same channel. Scale bar, 2 μm. (C) Uninucleate cells as in (B) were analyzed for Cdc13 signal at single (interphase) or separated (mitotic) SPBs. The mean and SD of 3 replicates are shown. Uninucleate population n > 200 cells per replicate; total n = 824 total for Cdc13^WT^ and 954 for Cdc13^HPM^. (D) Cell lengths of the entire uninucleate population and of cells with a Cdc13-sfGFP SPB focus from one replicate of (C). Error bars represent median, with whiskers delimiting the 25^th^ and 75^th^ percentiles. n = 212 total for Cdc13^WT^ and 214 for Cdc13^HPM^. (E and F) Cell length compared to presence of Plo1-mCherry and (E) Cdc13^WT^-sfGFP or (F) Cdc13^HPM^-sfGFP foci at the SPB. Endogenous Cdc13 is still expressed. Data are pooled from 3 replicates; mean cell length per cohort is plotted against % of cells within that cohort with Plo1-mCherry or Cdc13-sfGFP foci at the SPB. n ≥ 89 cells per cohort. Total n = 899 cells for Cdc13^WT^ and 903 cells for Cdc13^HPM^. (G) Cell length compared to Cdc13^HPM^-sfGFP SPB foci in strains that accelerate the accumulation of Polo kinase at the SPB and in a wild-type background. Data are pooled from 3 replicates and sorted into 1-μm bins. Data are given as percentage of cells in a given bin with a Cdc13^HPM^-sfGFP focus. n > 100 cells per replicate per strain. Total n = 310 cells for cut12.s11, 350 cells for cut12.T75DT78D, 244 cells for plo1.S402E, and 332 cells for wild-type (WT) cells. (H) Percentage of cells with Cdc13-sfGFP foci when released into mitosis in the presence of the temperature-sensitive *plo1-24c* allele. *cdc2*^*as*^*plo1-24c* cells carrying either Cdc13^WT^-sfGFP or Cdc13^HPM^-sfGFP were arrested in G2 by the addition of 1 μM 1-NmPP1 for 3.5 h. Cells labeled 36°C were shifted to the *plo1-24c* restrictive temperature 90 min before washing out 1-NmPP1 to release into mitosis. Cells were imaged 6 min after release from the 1-NmPP1 block. The mean and SD of 3 replicates are shown. n > 75 cells per replicate per strain with a minimum of 225 cells analyzed in total. See also Figure S2 and Table S2.

Although cells driven by Cdc13^HPM^ alone could undergo S phase, they arrested in G2 and could not complete the cell cycle (Figure 2A). As cyclin fragments encompassing the hydrophobic patch have been implicated in cyclin localization to the centrosome and mutations to the hydrophobic patch of the budding yeast cyclin Clb2 alter its localization to the bud neck, we investigated whether the Cdc13^HPM^ phenotype was due to altered Cdc13^HPM^ localization [18, 31, 32]. Wild-type Cdc13 is found in the nucleus, is visibly enriched at the SPB in G2, and decorates the mitotic spindle during mitosis [33]. Using exogenous Cdc13^HPM^-sfGFP [34], we observed that Cdc13^HPM^ localized to the nucleus but, in contrast to Cdc13^WT^, its localization to the SPB was delayed and only appeared enriched at the SPB in the longest cells in the population (Figures 2B–2D). In fission yeast, cell length reflects cell cycle position, and therefore, to test whether this delayed localization was due to progression to a late cell cycle stage or to increased Cdc13^HPM^ expression, cells were arrested in G2 and allowed to accumulate Cdc13^HPM^. Despite Cdc13^HPM^ accumulation, these cells still failed to form Cdc13^HPM^ SPB foci (Figure S2). We next examined more precisely when Cdc13^HPM^ SPB foci arose during the cell cycle by using Polo kinase SPB foci as a marker of mitotic entry [35]. Cdc13^WT^ was observed at the SPB before Polo (Figure 2E), but the temporal order of Polo and Cdc13 appearance was reversed in Cdc13^HPM^ cells, with cyclin foci only appearing after Polo foci, establishing that Cdc13^HPM^ foci are a mitotic phenomenon (Figure 2F). Given that Cdc13^HPM^ cells do not execute mitosis when endogenous Cdc13 is repressed, Cdc13^HPM^ foci are only visible because of a functional, endogenous copy of Cdc13 also present in the cells.

To investigate whether this mitotic Cdc13^HPM^ localization was linked to Polo SPB localization, we accelerated Polo SPB localization using either mutations in the SPB component Cut12 that reduce the CDK activity threshold for Polo foci formation (Cut12.s11 and Cut12^T75D,T78D^) [20, 35] or activating mutations in Polo itself (Plo1^S402E^) [36]. Both of these methods accelerated the SPB localization of Cdc13^HPM^, suggesting that mitotic Cdc13^HPM^ foci are downstream of Polo kinase activation at the SPB (Figure 2G). We then tested whether Polo kinase activity was necessary for mitotic Cdc13^HPM^ localization to the SPB by arresting cells in G2 using 1-NmPP1 in a Plo1 temperature-sensitive background [37]. After ablating Polo activity, 1-NmPP1 was withdrawn and cells released into mitosis. Cdc13^HPM^ foci appeared in far fewer cells when compared to cells released from the permissive temperature or cells expressing Cdc13^WT^, demonstrating that Cdc13^HPM^ mitotic SPB localization is dependent on Polo activity (Figure 2H). Cdc13^WT^ SPB foci in mitosis were not compromised by the inactivation of Polo kinase, suggesting that wild-type Cdc13 localizes to the SPB by both Polo and hydrophobic-patch-dependent mechanisms in mitosis. We conclude there are two temporally distinct mechanisms to localize Cdc13-CDK at the SPB—a hydrophobic-patch-dependent mechanism acting in G2 and a Polo-kinase-dependent mechanism acting at mitotic onset. Without a functional hydrophobic patch (and therefore G2 Cdc13-CDK SPB localization), cells cannot progress into mitosis.

### Loss of Centrosomal Cyclin-CDK Results in Impaired Global Mitotic Phosphorylation

**Figure 3:**
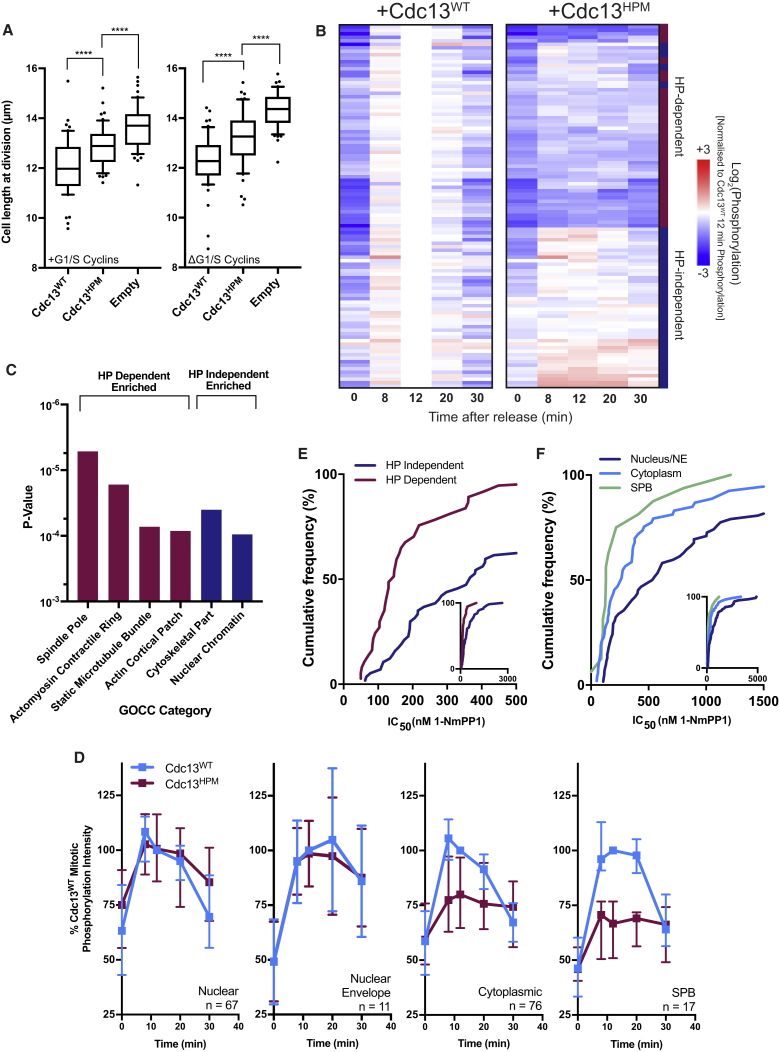
Loss of Centrosomal Cyclin-CDK Results in Impaired Global Mitotic Phosphorylation (A) Cell length measurements of either wild-type (left, +G1/S cyclins) or Δ*cig1* Δ*cig2* Δ*puc1* (right, ΔG1/S cyclins) cells expressing either exogenous Cdc13^WT/HPM^ or with no additional Cdc13 construct. p < 0.0001 for all comparisons using unpaired Student’s t test with Welch’s correction. Box is delimited by 25^th^ and 75^th^ percentiles and shows the mean. Whiskers delimit the 10th to 90th percentiles. n > 50 cells per condition. (B) Heatmap of mitotic CDK phosphosites after mitotic release. Each row represents a single CDK phosphosite. All measurements are normalized to phosphorylation intensity of the phosphosite at 12 min after release in the Cdc13^WT^ condition, and therefore, all Cdc13^WT^ measurements are 0 at this time point (this time point represents maximum mitotic phosphorylation). n = 105 phosphosites. Only sites that were found in all time points across both conditions are shown. Sites shown are hierarchically clustered (see STAR Methods). Right-hand bar represents sites that encompasses HP-dependent (red) and HP-independent (blue) categories. (C) Gene enrichment analysis of mitotic CDK phosphosites, showing p values obtained from Gene Ontology cellular compartment enrichment analysis (see STAR Methods). Analysis was conducted using Fisher’s exact test, using false discovery rate correction. (D) Phosphosite phosphorylation profiles for substrates considered to be either localized to the nucleus, SPB, nuclear envelope, or cytoplasm (see STAR Methods). Median phosphorylation values are plotted as a percentage of phosphorylation at t = 12 for Cdc13^WT^ phosphosites. Error bars represent the interquartile range; n numbers represent number of phosphosites detected at a given location. (E) Cumulative frequency curves of HP-dependent and HP-independent phosphosites against their average IC_50_ to 1-NmPP1 inhibition of CDK [5]. 0–400 nM 1-NmPP1 is shown on x axis; inset shows entire range of data (0–3,000 nM 1-NmPP1). One data point is beyond axis limits but included in statistical analysis. Mann-Whitney rank comparison p < 0.0001. (F) Cumulative frequency of differentially localized phosphosites against their average IC_50_ to 1-NmPP1 inhibition of CDK [5]. 0–1,500 nM 1-NmPP1 is shown on x axis; inset shows entire range of data (0–5,000 nM 1-NmPP1). 3 data points are beyond axis limits but included in statistical analysis. Difference between nucleus/NE versus cytoplasm: p < 0.01. Difference between nucleus/NE versus SPB: p < 0.001. Both are calculated by Mann-Whitney rank comparison. For determination of substrate location, see STAR Methods. See also Figures S3 and S4 and Table S2. See Table S1 for phosphoproteomic data.

Cdc13^HPM^ cannot execute mitosis but, when expressed with endogenous Cdc13, can accelerate mitotic entry (Figure 3A), suggesting that Cdc13^HPM^ is capable of some G2/M phosphorylation events. To identify Cdc13^HPM^ phosphorylation events *in vivo*, we used quantitative phosphoproteomics. Cells with repressed endogenous Cdc13 were synchronized in G2 and then released into mitosis while expressing either Cdc13^WT^ or Cdc13^HPM^ (Figure S3A). Cdc13^WT^-expressing cells progressed through mitosis; however, Cdc13^HPM^-expressing cells did not (Figure S3B). No notable changes in the proteome were observed, but there were significant changes in the phosphoproteome (Figure S3C). A total of 157 previously characterized CDK phosphosites were identified (Table S1). Analysis of the 52 sites phosphorylated early in the cell cycle showed that Cdc13^HPM^ could phosphorylate them all to levels similar to Cdc13^WT^ (Figure S3D), including proteins involved in S phase, such as Orc1, Mcm10, and Sld2. Of the 105 mitotic phosphosites that became phosphorylated later in the cell cycle, 57 phosphosites, corresponding to 50 proteins, could not be phosphorylated by Cdc13^HPM^-CDK to mitotic wild-type levels and were classed as hydrophobic patch (HP) dependent. The remaining 48 phosphosites, located on 38 proteins, could be phosphorylated to wild-type levels and were classed as HP independent (Figure 3B). HP-dependent substrates were enriched for SPB localization, with 12 of these proteins having previously been observed at the SPB (Figure 3C).

To investigate this further, we grouped substrates according to their reported subcellular localization. We observed that phosphorylation by Cdc13^HPM^-CDK displayed a relationship with cellular compartment, as SPB and cytoplasmic substrates cannot be phosphorylated to wild-type levels, whereas nuclear and nuclear-envelope-localized substrates are equally phosphorylated by Cdc13^WT^-CDK and Cdc13^HPM^-CDK (Figure 3D). We also observed a relationship between HP dependency and the substrate CDK-activity threshold. We previously defined substrate-specific CDK-activity thresholds by the concentration of a CDK inhibitor (1-NmPP1) that results in 50% of maximal phosphorylation (IC_50_) [5]. Upon examining Cdc13^HPM^ phosphorylation (Figure 3B), we see that HP-dependent substrates tend to have lower IC_50_ values than HP-independent substrates, meaning that HP-dependent substrates, on average, require higher CDK activity levels to become phosphorylated (Figure 3E). Accordingly, we also see that substrates at the SPB and in the cytoplasm also tend to have lower IC_50_ values (Figure 3F).

No obvious relationship between maximum phosphorylation reached in the Cdc13^HPM^ condition and the R/KxL content of a given substrate was observed. We also tested for a relationship with LxF content, as the budding yeast cyclin Clb2 has been found to bind this motif, but found none [27] (Figure S4). Although one functional docking site would be sufficient to lead to efficient phosphorylation, the existence of substrates with no suitable docking motifs that experience reduced phosphorylation with Cdc13^HPM^ (Figures S4D and S4E) suggests that the disruption of direct docking with the hydrophobic patch is not responsible for the reduced phosphorylation of these substrates.

We conclude that Cdc13^HPM^-CDK substrate phosphorylation is reduced when compared to Cdc13^WT^-CDK in a location-dependent manner and also in a [1-NmPP1]-defined substrate-IC_50_-dependent manner. Previous work has shown that localization of CDK and its substrates is a critical aspect of CDK substrate phosphorylation [16, 23]. We further suggest that CDK localization to the SPB is a determining factor for phosphorylation of CDK substrates in the cytoplasmic compartment and on the SPB and that Cdc13-CDK localization to the SPB is important for generating the highest levels of CDK activity in these compartments needed for mitosis.

### The Hydrophobic Patch Directs Human Cyclin B1 to Centrosomes

**Figure 4:**
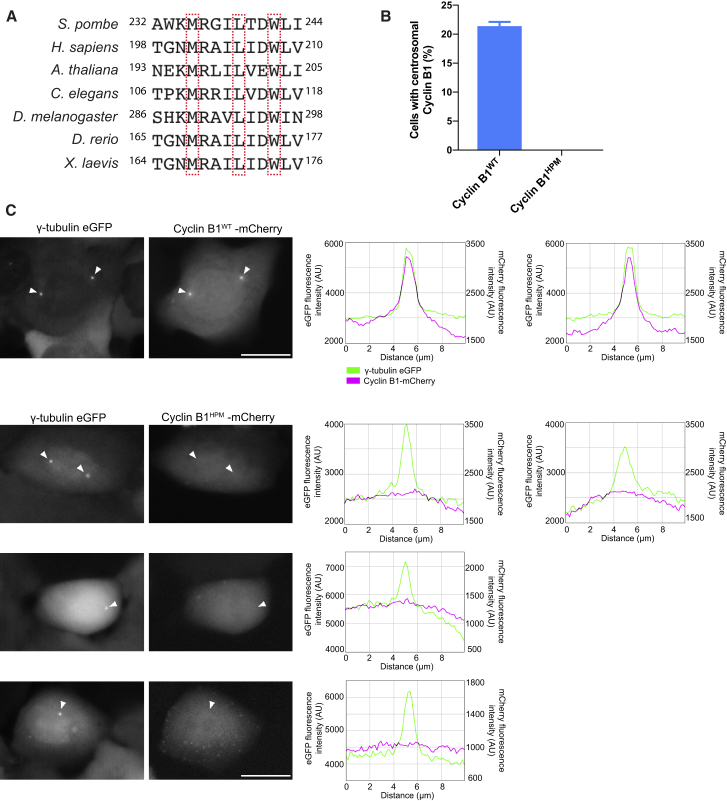
The Hydrophobic Patch Directs Human Cyclin B1 to Centrosomes (A) Alignment of major B-type cyclin sequences from evolutionarily distant eukaryotes. Boxed residues correspond to those mutated in this study to construct Cdc13^HPM^ and human cyclin B1^HPM^. (B) U2OS cells stably expressing γ-tubulin-EGFP (to mark the centrosome) were transfected with cyclin B1^WT/HPM^-mCherry. Cells from an asynchronous population were analyzed for centrosomal cyclin B1-mCherry signal. Mean and SD of 3 replicates are shown. n > 70 cells per replicate per condition. Total n = 294 cells for cyclin B1^WT^-mCherry and 287 cells for cyclin B1^HPM^-mCherry. (C) Representative cells as in (B) followed through division with time-lapse microscopy. Only the in-focus slice (judged by γ-tubulin-EGFP signal) is shown. All frames are the first time point with nuclear cyclin B1-mCherry indicating mitotic entry. Arrows show position of prominent γ-tubulin-EGFP foci, co-localization with cyclin B1^WT^, and lack of co-localization with cyclin B1^HPM^. Graphs show average pixel intensity of a line 3 pixels in height drawn through the centrosome. Scale bars, 20 μm. See also Videos S1 and S2.

The hydrophobic patch docking region is conserved in B-type cyclins in a wide range of eukaryotes (Figure 4A). It has previously been observed that cyclin B1 fragments encompassing the hydrophobic patch are able to localize to the centrosome and that CDK binding is not necessary for the centrosomal localization of cyclin B1 [18]. Therefore, we sought to determine whether the hydrophobic patch of cyclin B1 is responsible for targeting it to the centrosome in mammalian cells. We transiently transfected cyclin B1-mCherry constructs into human U2OS cells expressing γ-tubulin-EGFP. Cyclin B1^WT^-mCherry localized at centrosomes in a large fraction of cells as previously reported [38], but this localization was lost with cyclin B1^HPM^ (Figures 4B and 4C; Videos S1 and S2). Although, like Cdc13^HPM^, the interphase localization of cyclin B1 to the centrosome was lost in the cyclin B1^HPM^ condition, unlike Cdc13^HPM^, there appeared to be no cyclin B1^HPM^ localization to centrosomes in mitosis. This suggests that the centrosomal localization function of the hydrophobic patch is conserved between Cdc13 and cyclin B1.

Video S1. Time Lapse of Cyclin B1^WT^-mCherry, Related to Figure 4 U2OS cell stably expressing γ-tubulin-eGFP and transiently transfected with Cyclin B1^WT^-mCherry followed through division with time-lapse microscopy. Top: γ-tubulin-eGFP; bottom: Cyclin B1^WT^-mCherry. Images were taken every 10 minutes. Video shows the most in-focus slice of a z stack, judged by γ-tubulin-eGFP signal. Scale bar, 20μm.

Video S2. Time Lapse of Cyclin B1^HPM^-mCherry, Related to Figure 4 U2OS cell stably expressing γ-tubulin-eGFP and transiently transfected with Cyclin B1^HPM^-mCherry followed through division with time-lapse microscopy. Top: γ-tubulin-eGFP; bottom: Cyclin B1^HPM^-mCherry. Images were taken every 10 minutes. Video shows the most in-focus slice of a z stack, judged by γ-tubulin-eGFP signal. Scale bar, 20μm.

### Conclusions

We have shown that the hydrophobic patch on the mitotic cyclin Cdc13 is not required for cells to undergo S phase *in vivo* but is required to target Cdc13 to the SPB and for the complete execution of mitosis. In the absence of Cdc13-CDK localization to the SPB during G2, roughly half of all detected mitotic CDK phosphosites are not efficiently phosphorylated. These correspond to substrates at the SPB and in the cytoplasm and are also the substrates that require the highest levels of CDK activity to become phosphorylated. This suggests that correctly regulated Cdc13-CDK localization to the SPB during G2/M is required to reach the upper level of CDK activity at the SPB and in the cytoplasmic compartments necessary to phosphorylate these substrates and enter mitosis. These results suggest that, although both S-phase and M-phase cyclins use their docking regions to refine their substrate selection, S-phase cyclins primarily use the hydrophobic patch to directly bind to substrates, whereas we suggest that M-phase cyclins primarily use the hydrophobic patch to increase *in vivo* concentrations of cyclin-CDK in certain cellular compartments to aid the phosphorylation of otherwise poor CDK substrates.

The hydrophobic patch of M-phase cyclins was thought to be conserved due to its role in mediating localization of mitotic cyclin-CDK [18], and here, we show that this could be due to its effects on compartmentalized substrate phosphorylation. It is likely that docking motifs of a SPB component interact with the Cdc13 hydrophobic patch, governing Cdc13 localization in G2, and that this SPB enrichment allows the phosphorylation of other HP-dependent sites, even if they are not on R/KxL-containing proteins. Finally, the conservation of the cyclin B hydrophobic patch, and its role in centrosomal localization in human cells, furthers the view that the centrosome is a major mitotic signaling hub [16, 39, 40] and suggests that the hydrophobic patch centrosomal targeting mechanism and global mitotic phosphorylation regulation may be a conserved element in mitotic cell cycle control throughout eukaryotes.

## STAR★Methods

### Key Resources Table

**Table:** 

REAGENT or RESOURCE	SOURCE	IDENTIFIER
**Antibodies**		

Mouse monoclonal anti-Cdc13	Abcam	Cat# 6F11/2; RRID: AB_297544
Rabbit polyclonal anti-Cdc2-Y15P	Cell Signaling Technologies	Cat# 9111; RRID: AB_331460
HRP-conjugated goat anti-mouse	AbD SeroTEC	Cat# STAR120P; RRID: AB_567024
HRP-conjugated donkey anti-rabbit	GE Healthcare	Cat# NA934; RRID: AB_772206

**Chemicals, Peptides, and Recombinant Proteins**		

Thiamine Hydrochloride	Sigma Aldrich	Cat# T4625
cOmplete mini Protease Inhibitor Cocktail	Sigma Aldrich	Cat# 11836153001
PhosSTOP phosphatase Inhibitor tablets	Sigma Aldrich	Cat# PHOSS-RO
Propidium iodide solution	Biotium	Cat# 40017
Lipofectamine 3000	Invitrogen	Cat# L3000001
Pierce Trypsin Protease, MS Grade	ThermoFisher	Cat# 90058
1-NmPP1	Toronto Research Chemicals	Cat# A603003

**Critical Commercial Assays**		

Dynabeads Protein A	ThermoFisher	Cat# 10002D
Dynabeads M-270 Epoxy	ThermoFisher	Cat# 14302D
TMT 10plex Isobaric Label Reagent Set 1 × 0.8 mg	ThermoFisher	Cat# 90110
Pierce TiO2 Phosphopeptide Enrichment Spin Kits	ThermoFisher	Cat# 88303
High-Select Fe-NTA Phosphopeptide enrichment kit	ThermoFisher	Cat# A32992
UltiMate 3000 HPLC System	ThermoFisher	Cat# 5041.0010
EASY-Spray C18 column, 75 mm x 50 cm	ThermoFisher	Cat# ES803
Orbitrap Fusion Lumos Tribrid Mass Spectrometer	ThermoFisher	Cat# IQLAAEGAAPFADBMBCX
BD LSRFortessa	BD Biosciences	Cat# 649225

**Deposited Data**		

The full mass spectrometry proteomics data obtained in this study have been deposited with the ProteomeXchange Consortium via the PRIDE partner repository	This Paper	PRIDE: PXD011987

**Experimental Models: Cell Lines**		

Human U2OS γ-Tubulin-eGFP cells	[41]	N/A

**Experimental Models: Organisms/Strains**		

All S. pombe strains used in this study are listed in Table S2	Lab Stocks and this paper	N/A

**Recombinant DNA**		

Plasmid: leu1Δ::Cdc13-sfGFP_HphR	This Paper	SBp24
Plasmid: leu1Δ::Cdc13(HPM)-sfGFP_HphR	This Paper	SBp25
Plasmid: leu1Δ::Cdc13_HphR	This Paper	SBp29
Plasmid: leu1Δ::Cdc13(HPM)_HphR	This Paper	SBp30
Plasmid: CCNB1-mCherry	[42]	Addgene plasmid # 1958
Plasmid: CCNB1(HPM)-mCherry	This Paper	ERp12

**Software and Algorithms**		

FlowJo v10.1	FlowJo	https://www.flowjo.com/
Prism v7.0c	GraphPad	https://www.graphpad.com/scientific-software/prism/
ImageJ v1.50c	ImageJ	https://imagej.nih.gov/ij/
Perseus v1.4.0.2	Perseus	https://maxquant.net/perseus/
MaxQuant v1.5.0.13	MaxQuant	https://www.maxquant.org/
Ilastik v1.3.0	ilastik	https://www.ilastik.org/

### Lead Contact and Materials Availability

Further information and requests for resources and reagents should be directed to and will be fulfilled by the Lead Contact, Souradeep Basu (saz.basu@crick.ac.uk).

All strains, plasmids, and reagents generated in this study are available from the Lead Contact without restriction.

### Experimental Model and Subject Details

#### *S. pombe* genetics and cell culture

Experiments were conducted in yeast extract media supplemented with adenine, leucine, histidine and uracil to a final concentration of 0.15 g/L unless otherwise stated. Experiments involving the thiamine repressible promoter system were conducted in Edinburgh Minimal Media, all other experiments were carried out in yeast extract media with supplemented adenine, leucine, histidine and uracil as previously specified. Cells were maintained in exponential growth (between 2 × 10^6^ and 1 × 10^7^ cells/ml for all experiments. For experiments conducted in EMM, nitrogen and glucose were added separately and filter sterilized after addition. Cells were grown at 25°C unless stated otherwise.

In order to G1 arrest cells by nitrogen starvation, cells at a density of 2 × 10^6^ cells/ml were washed into EMM media lacking ammonium chloride or amino acid supplements for 16 hours. In cases where leucine auxotrophic strains were required to be arrested in G1, 0.05 g/L leucine was added to EMM media that otherwise lacked nitrogen. Cells were released by washing into EMM media containing ammonium chloride and all listed amino acid supplements. To shut off expression of the thiamine-repressible nmt41 promoter, thiamine hydrochloride dissolved in water and was added to 30 μM. Cell cycle arrest using *cdc2*^*as*^ was performed with the addition of 1 μM (for a G2 arrest) or 10 μM (for a G1 arrest) 1-NmPP1 for one cell cycle unless stated otherwise. Strains used in this work are listed in Table S2.

#### Human cell culture

Human female U2OS cells were maintained in Dulbecco’s modified Eagle’s medium (DMEM, GIBCO) containing 10% FBS, 50 U/ml penicillin and 50 μg/ml streptomycin at 37°C and 5% CO_2_. Transfection was performed using Lipofectamine 3000 reagent (Invitrogen) according to manufacturer’s instructions in Opti-MEM media (GIBCO). Cell lines were authenticated and confirmed as mycoplasma-free by the Cell Services science technology platform at the Francis Crick Institute.

### Method Details

#### Serial Dilution Assays

Cells were taken from a culture of exponentially growing cells at a density of 5 × 10^6^ cells/ml or from an indicated time point following release, which corresponds to the leftmost dilution of all dilution assays, followed by repeated 1:10 dilutions. 4 μL of cell suspension was plated for each spot.

#### Cell cycle progression determination

DNA content analyses were conducted with 0.4 mL of cell suspension of cell density > 3.78 × 10^6^ cells/ml fixed by addition of 0.91 mL of 100% ethanol to give a final concentration of 70% v/v ethanol suspension. Cells were kept on ice for > 30 minutes before pelleting at 12,000 rpm and resuspending into 50mM sodium citrate with 0.1 mg/ml RNase A for over 3 hours. DNA was then stained with propidium iodide by addition to 2 μg/ml before sonication. At least 10,000 cells were acquired per sample on a BD LSRFortessa flow cytometer. DNA content is shown on a linear scale after gating for single cells in FlowJo X.

To score for nuclear division and cell septation indices, 4 μL of cell suspension was heat fixed at 70°C before addition of DAPI to monitor DNA, and Calcofluor to monitor septum formation. Size at division was measured from live Calcofluor stained cells by measuring septated cells. For determination of these indices, samples were imaged on a Zeiss Axioskop, 63x/1.4 NA objective. All experiments involving cell cycle progression determination were repeated with similar findings.

#### Protein Extraction and Western Blotting

Protein was extracted from cell culture initially by quenching with 100% w/v ice-cold trichloroacetic acid to a final concentration of 10%. Cells were stored on ice for 20 minutes, pelleted at 3000 x g, and washed in acetone before storage at −80°C. After storage, pellets were resuspended in lysis buffer (8M Urea, 50 mM ammonium bicarbonate, 1x cOmplete mini EDTA-free protease inhibitor + 1x phosSTOP phosphatase inhibitor cocktail). Roughly 1.2 mL of 0.4 mm acid-washed glass beads were then added to suspensions, which were subject to three rounds of beating at 5.5 m/s for 30 s (FastPrep120). Cell debris was then pelleted at 16,000 x *g* for 5 minutes, and supernatant stored as whole-cell protein sample at −80°C.

Protein detection by western blotting was performed for: Cdc2-Y15P using 1:500 anti-Cdc2-Y15P (rabbit polyclonal) (#9111, Cell Signaling Technologies); or for Cdc13 using 1:500 anti-Cdc13 (mouse monoclonal) (6F11/2, ab10873, Abeam). Secondary antibodies: 1:25,000 horseradish peroxidase-conjugated donkey anti-rabbit (NA934, GE Healthcare) or 1:5000 goat anti-mouse (STAR120P, AbD SeroTEC). Signal was detected using SuperSignal

West Femto Maximum Sensitivity Substrate (34095, Life Technologies) and imaged on either an ImageQuant LAS 4000 or an Amersham Imager 600.

#### Tandem Mass Tag Proteomics

300 μg of each protein sample was reduced with 5 mM dithiothreitol (56°C, 25 min), alkylated with 10 mM iodoacetamide (room temperature, 30 min, dark), and quenched with 7.5 M DTT. Samples were then diluted with 50 mM HEPES to reduce the urea concentration to < 2 M, prior to trypsin digestion (37°C, overnight). Peptides were then acidified and desalted using a C_18_ SepPak under vacuum and dried. The samples were then labeled by the use of a Thermo Scientific TMT10plex Isobaric Label Reagent Set, 10 × 0.8 mg, as per manufacturer’s instructions. Following successful label and mixing checks, multiplexed samples were again desalted using a C_18_ SepPak. Phosphopeptide enrichment was completed using titanium dioxide (TiO_2_) beads: Dried peptide mixtures were re-suspended in 1 M glycolic acid + 80% acetonitrile + 5% trifluoroacetic acid, sonicated (10 min) and added to TiO_2_ beads (5:1 (w/w) beads:protein), the beads were washed using 80% acetonitrile + 1% trifluoroacetic acid followed by 10% acetonitrile + 0.2% trifluoroacetic acid, and dried under vacuum centrifugation. Flow-through fractions were retained for analysis of non-phosphorylated peptides. Phosphopeptides were eluted from the beads by adding 1% ammonium hydroxide followed by 5% ammonium hydroxide. Phosphopeptides and non-phosphopeptides were both fractionated by the use of a Pierce High pH Reversed-Phase Fractionation Kit and each eluted fraction analyzed with a Thermo Fisher Orbitrap Fusion Lumos mass spectrometer coupled to an UltiMate 3000 HPLC system for on-line liquid chromatographic separation. Each run consisted of a 3 h gradient elution (75 μm × 50 cm C_18_ column). A technical repeat of the mass spectrometry data was performed, and similar results obtained.

#### Fluorescence microscopy

All live cell fluorescence microscopy was performed using a Nikon Ti12 inverted microscope with Perfect Focus System and Okolab environmental chamber, and a Prime sCMOS camera (Photometrics). The microscope was controlled with Micro-Manager v2.0 software (Open-imaging) [43]. Fluorescence excitation was performed using a SpectraX LED light engine (Lumencor) fitted with standard filters: 470/24 for imaging sfGFP/eGFP; and 575/25 for imaging mCherry/mRFP; with either a dual-edge ET-eGFP/mCherry dichroic beamsplitter, Chroma 59022bs, or a BrightLine® quad-edge dichroic beamsplitter, Semrock FF409-493-573-652. Emission filters were as follows: Chroma, ET - EGFP single-band bandpass filter ET525_50m for imaging sfGFP/eGFP; and Semrock, 641/75 nm BrightLine® single-band bandpass filter FF02_641_75 for imaging mCherry/mRFP. ImageJ software (NIH) was used to measure pixel intensity, adjust brightness and contrast and render maximum projection images [44].

For *S. pombe* experiments, images were taken using a 100X Plan Apochromat oil-immersion objective (NA 1.45) at 25°C. Cdc13-sfGFP and Polo-mCherry accumulation at the SPB was judged by eye. In strains in which an SPB marker (Sid4-mRFP) was present, any cells without Sid4-mRFP signal were excluded from analysis. Mean whole-cell fluorescence intensity measurements for *cdc2*^*as*^ G2-arrested cells were performed on maximum projection images. Cells were segmented based on brightfield images using Ilastik [45], and the masks applied to fluorescence images to measure the mean pixel intensity within the mask. Background autofluorescence was calculated by measuring the mean whole-cell fluorescence intensity of *cdc2*^*as*^ cells without an exogenous copy of Cdc13^WT/HPM^-sfGFP. This was found to be similar across all cell lengths, so the y-intercept value of a linear regression plotted through the data (rounded to the nearest 10 AU) was taken as the autofluorescence value. This was subtracted from all fluorescence intensity measurements.

For U2OS cell experiments, cells were seeded 24 hours before transfection in 35mm glass-bottom dishes (MatTek Corporation). At 24 hours after transfection, fluorescence imaging was performed with the environmental chamber heated to 37°C with 5% CO_2_ supply. For time-lapse, z stacks were acquired at 10-minute intervals. All imaging was performed using a 40X Plan Apochromat objective (NA 0.95). Cells with Cyclin B1-mCherry expression below 9000 AU were included for analysis; cells with expression higher than this were excluded as high levels of Cyclin B1 overexpression may result in its mislocalization. Cells with signal intensity below roughly 1000 AU were excluded as the signal was too close to background to distinguish Cyclin B1 localization, as were cells in which the centrosome was not in focus (judged by γ-tubulin-eGFP signal). Cyclin B1-mCherry accumulation at the centrosome was judged by eye and by checking for an overlapping peak of γ-tubulin-eGFP and Cyclin B1-mCherry pixel intensity on line scans. All microscopy experiments were repeated with similar results obtained.

### Quantification and Statistical Analysis

#### Mass Spectrometry Data Analysis

MaxQuant (version 1.5.0.13) was used for all data processing. The data was searched against a UniProt extracted *Schizosaccharomyces pombe* proteome FASTA file, amended to include common contaminants. Default MaxQuant parameters were used with the following adjustments: Phospho(STY) was added as a variable modification (for the phosphopeptide enriched samples), MaxQuant output files were imported into Perseus (version 1.4.0.2) for further data analysis. For the generation of hierarchically-clustered heatmaps, Perseus was used with Settings: Cluster rows, Euclidian distance, do not presuppose K-means. For all analysis, phosphosite intensities are normalized to the intensity of the phosphosite at 12 minutes in the wild-type condition. This is taken to be representative of near maximal mitotic phosphorylation. All other phosphosite intensities are then given relative to this value. For the analysis shown in Figure 3B, phosphosite intensities were log transformed, and represented as log change in phosphorylation intensity relative to the 12 minute wild-type time point, therefore all values in the 12 minute wild-type condition are zero. For all other figures, phosphorylation is given on a linear scale, with all 12 minute wild-type phosphorylation taken as 100%.

#### Gene Enrichment Analysis

Gene enrichment analysis for cellular compartment enrichment was conducted using online server located at: geneontology.org/page/go-enrichment-analysis. Analysis was conducted using Fisher’s Exact test, using False discovery rate correction. The P value given represents the probability of seeing the observed number of genes in the submitted list of genes annotated to a particular gene ontology term, given the proportion of the genes in the whole genome that are annotated to that gene ontology term. The closer to zero a p value is, the less likely the observed annotation of the particular GO term to a group of genes occurs by chance.

#### Determination of CDK substrate localization

To determine the localization of all detected CDK substrates, we manually annotated each phosphosite with localization data of its encompassing protein. To balance conflicting localization data, we adopted a linear annotation strategy that first prioritises approaches that visualize the protein of interest directly by fluorescence microscopy when labeled at its endogenous locus. If this data was not available, we then considered fluorescently tagged proteins expressed from exogenous nmt41 and nmt81 promoters, which we consider to be at an expression level that mirrors endogenous levels for a large number of proteins. If no data was available, we then moved to considering indirect experimental evidence of location, which includes ChIP assays, fractionated western blotting, and immunoprecipitation experiments with proteins of known localization. Finally, if all the above was not available, we relied on localization data from a previously published YFP-tagged genome-wide overexpression library [46]. All references used for protein localization are given in Table S1.

#### Statistical Tests

Student’s unpaired two-tailed t tests were used to compare conditions in all cases where the data were judged to adhere to a normal distribution. Normality was checked by the D’Agostino-Pearson omnibus normality test. If the results of this test judged the data to be non-normal, then the Mann-Whitney rank-comparison test was conducted instead. All statistical tests were conducted using GraphPad Prism. Sample sizes were not pre-determined, however for cell length measurements we aimed to count over 50 cells. All sample sizes and n-numbers are given in figure legends. Information regarding all box and whisker plots are also defined in all figure legends.

### Data and Code Availability

The full mass spectrometry proteomics data obtained in this study have been deposited with the ProteomeXchange Consortium via the PRIDE partner repository. The accession number for the mass spectrometry data reported in this paper is PRIDE: PXD011987.

## Supplemental Information

Document S1. Figures S1–S4

Table S1. Condensed Phosphoproteomic Dataset, Related to Figures 3, S3, and S4

Table S2. *S. pombe* Strain List, Related to Figures 1, 2, 3, S1, S2, S3, and S4

Document S2. Article plus Supplemental Information
