# Supplementary material for: The Hydrophobic Patch Directs Cyclin B to Centrosomes to Promote Global CDK Phosphorylation at Mitosis
Source: Curr Biol. 2020 Mar 9;30(5):883–892.e4. doi: 10.1016/j.cub.2019.12.053 (PMC7063568; doi:10.1016/j.cub.2019.12.053)
Supplement: Document S1. Figures S1–S4 [file mmc1.pdf]

Current Biology, Volume *30*

## Supplemental Information

# The Hydrophobic Patch Directs Cyclin B to Centrosomes to Promote Global CDK Phosphorylation at Mitosis

**Souradeep Basu, Emma L. Roberts, Andrew W. Jones, Matthew P. Swaffer, Ambrosius P. Snijders, and Paul Nurse**

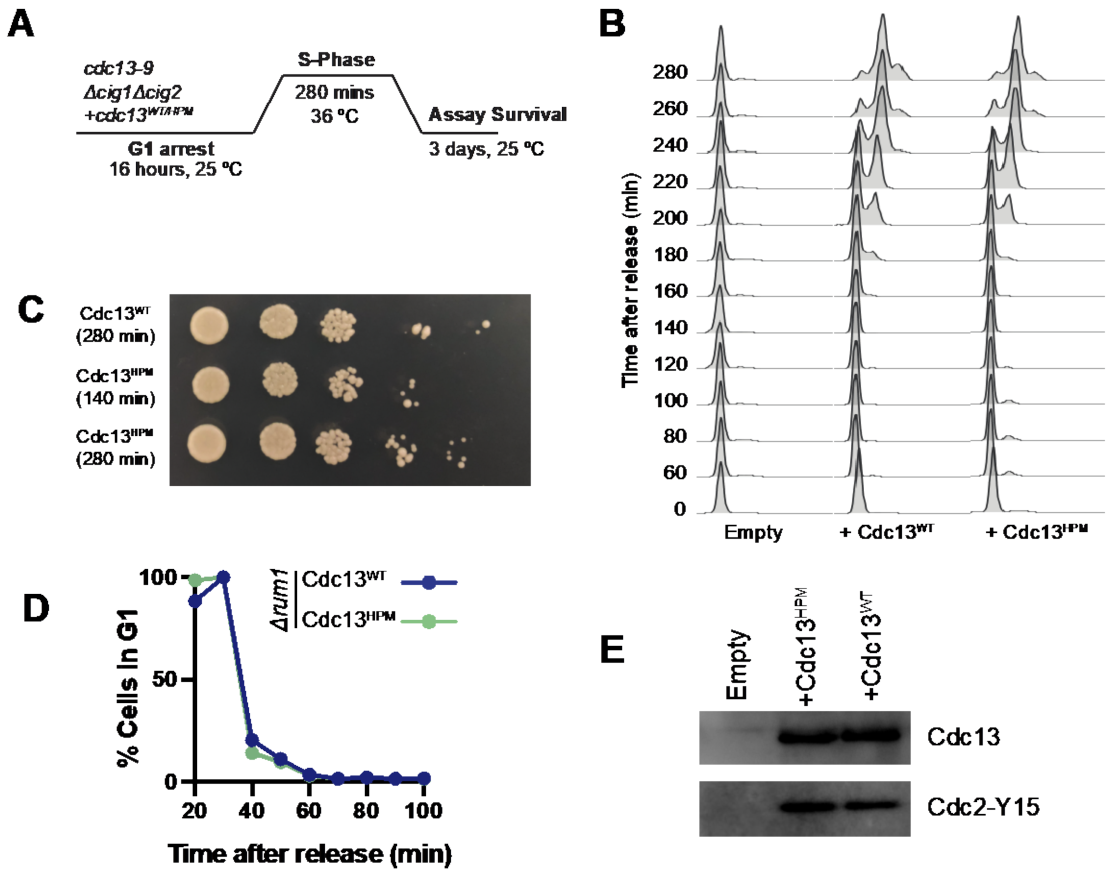

A
cdc13-9
Δcig1Δcig2
+cdc13WT/HPM
S-Phase
280 mins
36 °C
Assay Survival
G1 arrest
16 hours, 25 °C
3 days, 25 °C
B
Time after release (min)
280
260
240
220
200
180
160
140
120
100
80
60
0
Empty
+ Cdc13WT
+ Cdc13HPM
C
Cdc13WT
(280 min)
Cdc13HPM
(140 min)
Cdc13HPM
(280 min)
D
% Cells In G1
Δrum1
Cdc13WT
Cdc13HPM
100
50
0
20
40
60
80
100
Time after release (min)
E
Empty
+Cdc13HPM
+Cdc13WT
Cdc13
Cdc2-Y15

**Figure S1. Cdc13$^{HPM}$ executes a functional S-phase, Related to Figure 1.**

A – Experiment outline for panels (B) and (C) for testing survival after Cdc13$^{HPM}$ dependent S-phase. Cells were initially arrested in EMM lacking nitrogen for 16 hours before re-feeding with ammonium chloride (see STAR Methods). Cells were shifted to 36 $^{o}$C upon release, in order for them to conduct S-phase at the *cdc13-9* restrictive temperature. Serial dilution assays were conducted after shifting cells back to 25 $^{o}$C after S-phase completion to check if DNA replication had resulted in viable cells.

B – Flow cytometry profiles for cells after S-phase release at *cdc13-9* restrictive temperature of 36 $^{o}$C. Cells without an exogenous Cdc13 remain arrested in G1. 10,000 cells per timepoint were collected (see STAR Methods).

C – Cells were taken from the timecourse in (B) and checked for viability after 3 days of growth. These cells have undergone S-phase at the *cdc13-9* restrictive temperature, and therefore executed DNA replication using their exogenous Cdc13. Cells taken at 280 minutes following release have executed a Cdc13$^{HPM}$ S-phase, whereas cells taken 140 minutes after release can also rely on endogenous Cdc13, as they are shifted back to 25$^{o}$C before S-phase. Cells were plated onto YE4S at the *cdc13-9* permissive temperature of 25$^{o}$C.

D – The same cells used in Figure 1C, *cdc2(as) cdc13*-Switch Off *Δcig1 Δcig2* cells +*cdc13$^{WT/HPM}$,* were combined with a *rum1* deletion. Cells were arrested with 1 μM 1-NmPP1 for 1.5 cell cycles, and then washed of 1-NmPP1 to release cells into mitosis and the subsequent G1 and S-phase. Thiamine was added 1 hour before release from mitosis, and cells were kept in thiamine following mitosis. The S-phase following release from 1-NmPP1 inhibition was monitored using flow cytometry, with 10,000 events collected per time point (see STAR Methods).

E – Cdc2 Y15 phosphorylation with endogenous Cdc13 repressed, in the presence of exogenous Cdc13$^{WT}$ or Cdc13$^{HPM}$. In order to assay the sensitivity of Cdc13$^{WT}$ or Cdc13$^{HPM}$-CDK to Wee1 alone, *cdc2(as) cdc13*-Switch Off *Δcig1 Δcig2* cells +*cdc13$^{WT/HPM}$* (right and middle column, respectively) or + no insert (left column) were arrested in G2 using 1.5 μM 1-NmPP1 for 3.5 hours, with thiamine added 1 hour before release to repress endogenous Cdc13. Cells were then released into mitosis, and re-blocked with 10μM 1-NmPP1 for 2.5 hours. Cells were then released into DMSO and collected. Endogenous Cdc13 is completely degraded (left column) and therefore does not contribute to Cdc2 Y15 phosphorylation.

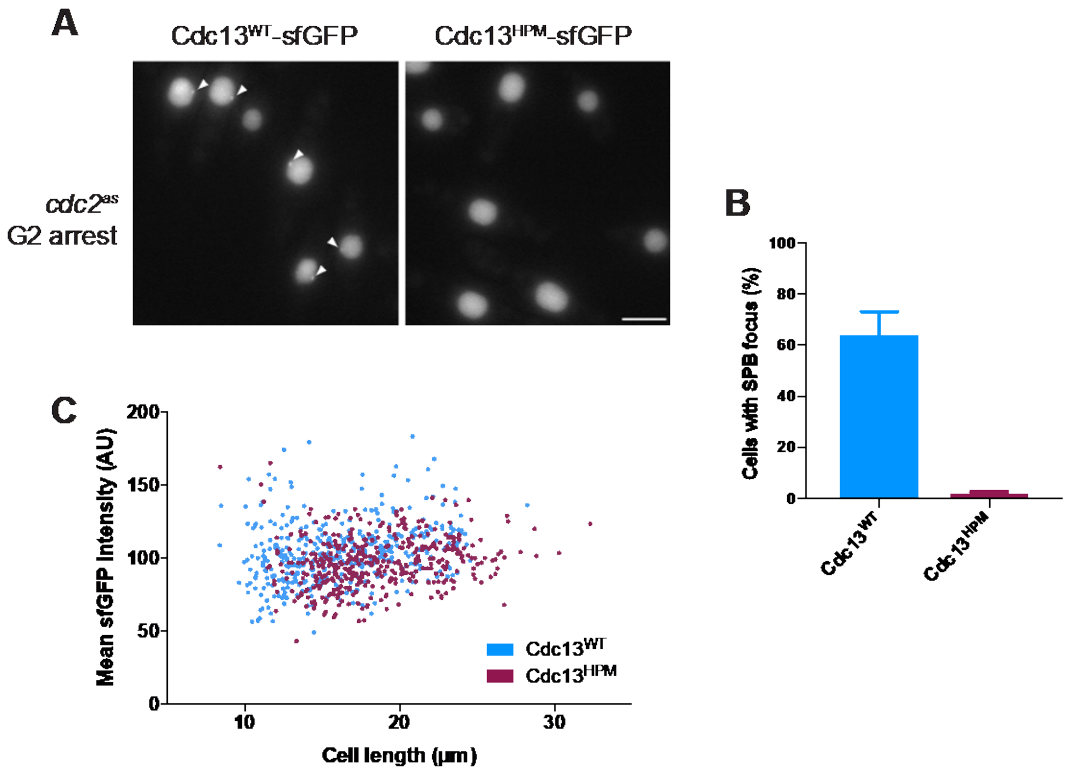
A
Cdc13WT-sfGFP
Cdc13HPM-sfGFP
cdc2as
G2 arrest
B
Cells with SPB focus (%)
100
80
60
40
20
0
Cdc13WT
Cdc13HPM
C
Mean sfGFP Intensity (AU)
200
150
100
50
0
10
20
30
Cell length (μm)
Cdc13WT
Cdc13HPM

**Figure S2. Cdc13$^{HPM}$-sfGFP does not accumulate at the SPB in a G2 arrest, Related to Figure 2.**

A – Representative maximum projection images of *cdc2(as)* cells arrested in G2 for 1 cell cycle containing an exogenous copy of either Cdc13$^{WT}$-sfGFP or Cdc13$^{HPM}$-sfGFP. The endogenous Cdc13 is expressed but not fused to a fluorophore. Arrows indicate Cdc13-sfGFP foci. The pixel range shown is the same for both Cdc13$^{WT}$ and Cdc13$^{HPM}$. Scale bar = 5 μm.

B – The percentage of G2-arrested *cdc2(as)-M17* cells with Cdc13-sfGFP foci. n > 250 cells per condition per replicate. The mean and SD of 3 replicates are shown. Total n = 882 cells for Cdc13$^{WT}$ and 794 cells for Cdc13$^{HPM}$.

C – Mean whole-cell fluorescence intensity of Cdc13$^{WT}$-sfGFP and Cdc13$^{HPM}$-sfGFP in G2 arrested cells, plotted against cell length, from one replicate of panel B. The mean value of background autofluorescence is removed. n = 428 cells for Cdc13$^{WT}$ and 410 cells for Cdc13$^{HPM}$.

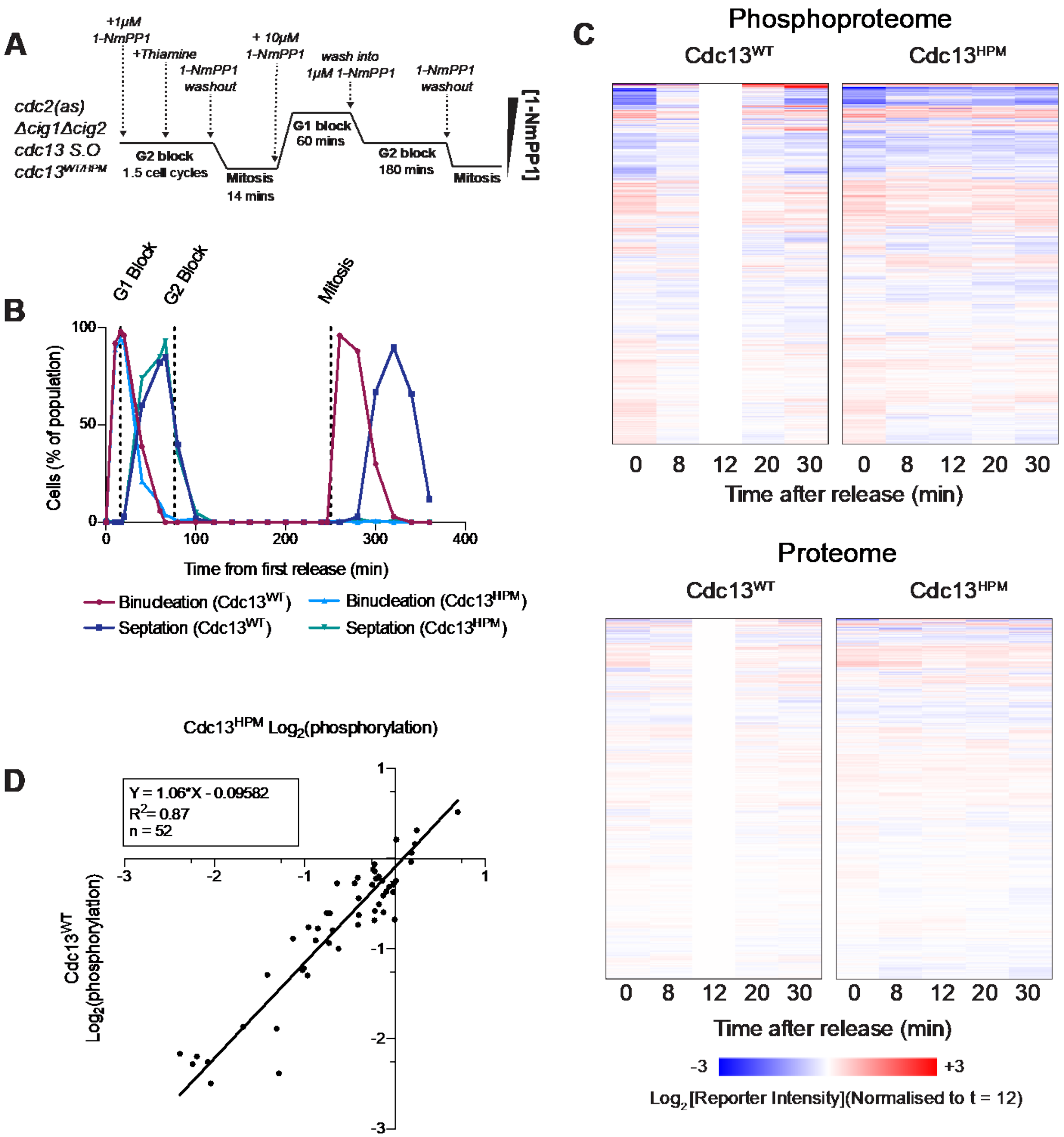

A
+1μM 1-NmPP1
+Thiamine
1-NmPP1 washout
+ 10μM 1-NmPP1
wash into 1μM 1-NmPP1
1-NmPP1 washout
cdc2(as)
Δcig1Δcig2
cdc13 S.O
cdc13WT/HPM
G2 block
1.5 cell cycles
Mitosis
14 mins
G1 block
60 mins
G2 block
180 mins
Mitosis
[1-NmPP1]
B
G1 Block
G2 Block
Mitosis
Cells (% of population)
100
50
0
0
100
200
300
400
Time from first release (min)
Binucleation (Cdc13WT)
Binucleation (Cdc13HPM)
Septation (Cdc13WT)
Septation (Cdc13HPM)
C
Phosphoproteome
Cdc13WT
Cdc13HPM
0
8
12
20
30
Time after release (min)
Proteome
Cdc13WT
Cdc13HPM
0
8
12
20
30
Time after release (min)
-3
+3
Log2[Reporter Intensity](Normalised to t = 12)
D
Cdc13HPM Log2(phosphorylation)
Y = 1.06*X - 0.09582
R2= 0.87
n = 52
-3
-2
-1
1
1
-1
-2
-3
Cdc13WT Log2(phosphorylation)

**Figure S3. Cdc13$^{HPM}$ can efficiently phosphorylate non-late CDK substrates, Related to Figure 3.**

A – Experiment outline for Figure 3 and S3. Cells are blocked in G2 initially using 1 μM of the ATP analogue 1-NmPP1. One hour before release from 1-NmPP1, thiamine was added to repress endogenous Cdc13 (see STAR Methods). Cells are then allowed to progress through one mitosis in the presence of thiamine, and subsequently re-blocked with for 60 mins using 10 μM inhibitor and thiamine 14 minutes after release into mitosis. Cells are then washed into 1 μM inhibitor and thiamine to arrest in G2 for 180 minutes before final mitotic release into no inhibitor. Time-points are taken following second mitotic release.

B – Septation and binucleation indices for +Cdc13$^{WT}$ and +Cdc13$^{HPM}$ release relating to panels in Figure 3 and S3. 100 fixed cells per time point were counted. See STAR Methods for details.

C – Lower panels: Proteome dataset heatmap encompassing 2757 individual proteins. Upper panels: Phosphoproteome dataset heatmap encompassing 3835 phosphosites. Heatmap was clustered according to Euclidian clustering in Perseus. All individual samples were normalised to reporter intensity for 12 minutes for the wild-type condition. See STAR Methods for further information.

D – Comparison of relative phosphorylation for non-late phosphosites (as defined in [S1]) at t = 0 for both Cdc13$^{HPM}$ and Cdc13$^{WT}$ releases. Statistics are given in panel.

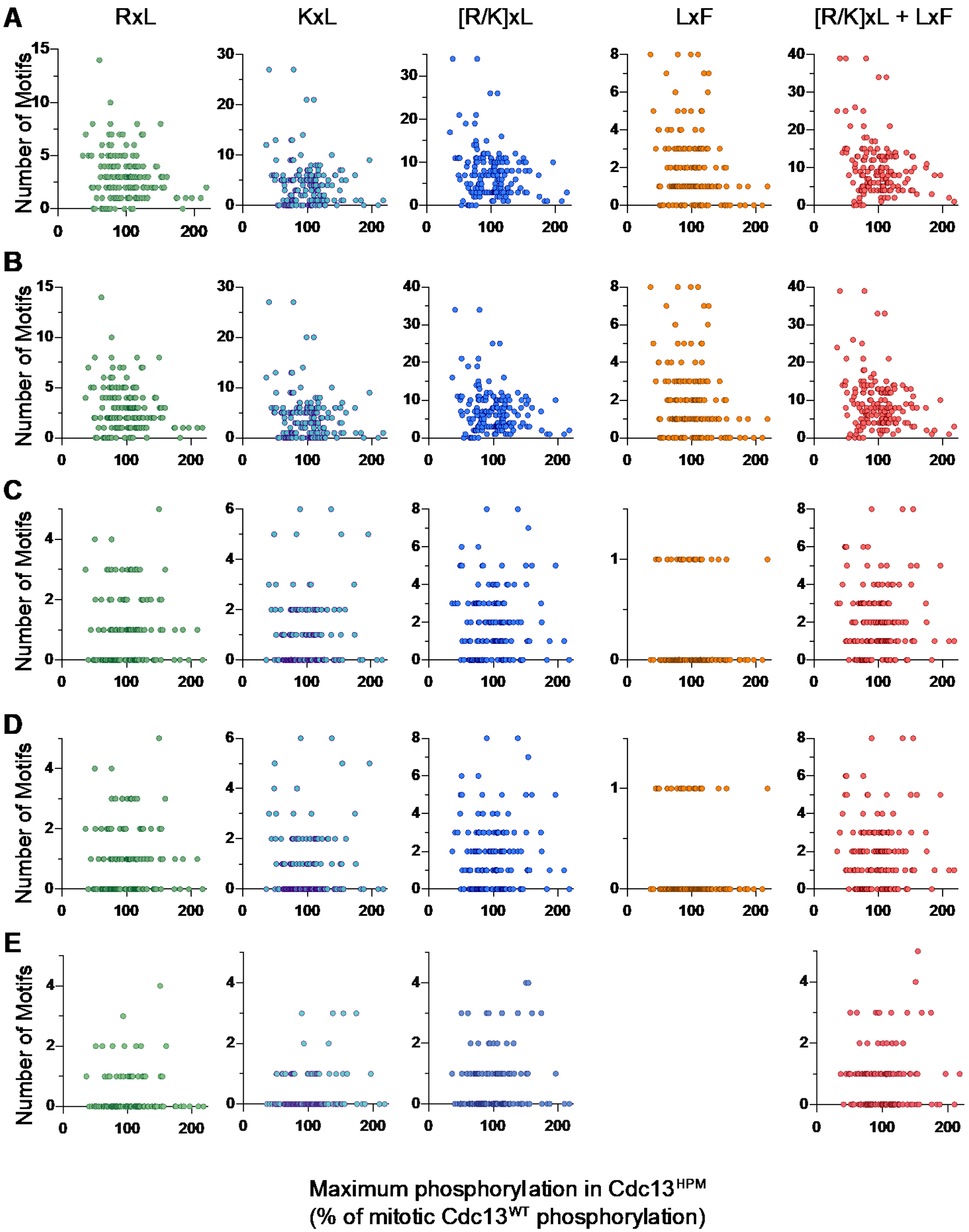
A
RxL
KxL
[R/K]xL
LxF
[R/K]xL + LxF
B
C
D
E
Number of Motifs
Maximum phosphorylation in Cdc13^HPM
(% of mitotic Cdc13^WT phosphorylation)

**Figure S4. Cdc13$^{HPM}$ dependent phosphorylation is not correlated with [R/K]xL or LxF content, Related to Figure 3.**

Analysis of relationship between motif numbers of phosphosite-encompassing proteins and maximum phosphorylation achieved by Cdc13$^{HPM}$ (given as a comparison with phosphorylation 12 minutes after release in the Cdc13$^{WT}$ condition). No data presented were suitable for analysis by linear regression as no r-square values above 0.066 were obtained when linear regression was attempted. Motifs analysed are given above each column of graphs. Raw data present in Supplementary Table 1.

A – Analysis of phosphorylation vs. raw numbers of motifs present in the protein that encompasses the phosphosite in question.

B – Analysis of phosphorylation vs. numbers of motifs present in the protein that encompasses the phosphosite in question. In addition, filtering was applied to exclude motifs that were closer than 12 amino acids in primary sequence to the phosphosite in question. This filtering was applied as the minimum distance between the hydrophobic patch and the active site of CDK has been mapped to be at least 12 amino acids in length for *S. cerevisiae* Clb2-Cdc28 and human Cyclin A-Cdk2 [S2, S3].

C – Analysis of phosphorylation vs. numbers of motifs present in the protein that encompasses the phosphosite in question. In addition, filtering was applied to exclude motifs that were not present in disordered regions of the protein, as motifs that interact with the hydrophobic patch are thought to be generally disordered. Disorder was checked using the IUPred2 server, with an average score of 0.5 across the motif being considered disordered.

D – Analysis of phosphorylation vs. numbers of motifs present in the protein that encompasses the phosphosite in question with filtering in both (B) and (C) applied.

E – Analysis of phosphorylation vs. numbers of motifs present in the protein that encompasses the phosphosite in question with filtering in both (B) and (C) applied. In addition, RxL and KxL motifs analysed were restricted to those that possessed the full R/KxL motif with a C-terminal hydrophobic residue at either or both the +1 or +2 positions from the terminal leucine of the R/KxL motif ([R/K]-X-L-X{0,1}-[FYLIPRVM]). No extended motif was analysed for LxF. The [R/K]xL + LxF panel sums extended [R/K]xL motifs with all LxF motifs.

## Supplemental References


S1. Swaffer, M.P., Jones, A.W., Flynn, H.R., Snijders, A.P., and Nurse, P. (2016). CDK Substrate Phosphorylation and Ordering the Cell Cycle. Cell *167*, 1750-1761 e1716.

S2. Koivomagi, M., Ord, M., Iofik, A., Valk, E., Venta, R., Faustova, I., Kivi, R., Balog, E.R., Rubin, S.M., and Loog, M. (2013). Multisite phosphorylation networks as signal processors for Cdk1. Nat Struct Mol Biol *20*, 1415-1424.

S3. Takeda, D.Y., Wohlschlegel, J.A., and Dutta, A. (2001). A bipartite substrate recognition motif for cyclin-dependent kinases. J Biol Chem *276*, 1993-1997.

S4. Sutani, T., Yuasa, T., Tomonaga, T., Dohmae, N., Takio, K., and Yanagida, M. (1999). Fission yeast condensin complex: essential roles of non-SMC subunits for condensation and Cdc2 phosphorylation of Cut3/SMC4. Genes Dev *13*, 2271-2283.

S5. Ikemoto, S., Nakamura, T., Kubo, M., and Shimoda, C. (2000). S. pombe sporulation-specific coiled-coil protein Spo15p is localized to the spindle pole body and essential for its modification. J Cell Sci *113 ( Pt 3)*, 545-554.

S6. Matsuyama, A., Arai, R., Yashiroda, Y., Shirai, A., Kamata, A., Sekido, S., Kobayashi, Y., Hashimoto, A., Hamamoto, M., Hiraoka, Y., et al. (2006). ORFeome cloning and global analysis of protein localization in the fission yeast Schizosaccharomyces pombe. Nat Biotechnol *24*, 841-847.

S7. Yamashita, A., Sato, M., Fujita, A., Yamamoto, M., and Toda, T. (2005). The roles of fission yeast ase1 in mitotic cell division, meiotic nuclear oscillation, and cytokinesis checkpoint signaling. Mol Biol Cell *16*, 1378-1395.

S8. Bestul, A.J., Yu, Z., Unruh, J.R., and Jaspersen, S.L. (2017). Molecular model of fission yeast centrosome assembly determined by superresolution imaging. J Cell Biol *216*, 2409-2424.

S9. Garcia, M.A., Vardy, L., Koonrugsa, N., and Toda, T. (2001). Fission yeast ch-TOG/XMAP215 homologue Alp14 connects mitotic spindles with the kinetochore and is a component of the Mad2-dependent spindle checkpoint. EMBO J *20*, 3389-3401.

S10. Toya, M., Iino, Y., and Yamamoto, M. (1999). Fission yeast Pob1p, which is homologous to budding yeast Boi proteins and exhibits subcellular localization close to actin patches, is essential for cell elongation and separation. Mol Biol Cell *10*, 2745-2757.

S11. Cruz, S., Munoz, S., Manjon, E., Garcia, P., and Sanchez, Y. (2013). The fission yeast cell wall stress sensor-like proteins Mtl2 and Wsc1 act by turning on the GTPase Rho1p but act independently of the cell wall integrity pathway. Microbiologyopen *2*, 778-794.

S12. Pollard, L.W., Onishi, M., Pringle, J.R., and Lord, M. (2012). Fission yeast Cyk3p is a transglutaminase-like protein that participates in cytokinesis and cell morphogenesis. Mol Biol Cell *23*, 2433-2444.

S13. Munoz, S., Manjon, E., and Sanchez, Y. (2014). The putative exchange factor Gef3p interacts with Rho3p GTPase and the septin ring during cytokinesis in fission yeast. J Biol Chem *289*, 21995-22007.

S14. Das, M., Wiley, D.J., Medina, S., Vincent, H.A., Larrea, M., Oriolo, A., and Verde, F. (2007). Regulation of cell diameter, For3p localization, and cell symmetry by fission yeast Rho-GAP Rga4p. Mol Biol Cell *18*, 2090-2101.

S15. Lopez-Aviles, S., Lambea, E., Moldon, A., Grande, M., Fajardo, A., Rodriguez-Gabriel, M.A., Hidalgo, E., and Aligue, R. (2008). Activation of Srk1 by the mitogen-

activated protein kinase Sty1/Spc1 precedes its dissociation from the kinase and signals its degradation. Mol Biol Cell *19*, 1670-1679.
S16. Kanda, Y., Satoh, R., Matsumoto, S., Ikeda, C., Inutsuka, N., Hagihara, K., Matzno, S., Tsujimoto, S., Kita, A., and Sugiura, R. (2016). Skb5, an SH3 adaptor protein, regulates Pmk1 MAPK signaling by controlling the intracellular localization of the MAPKKK Mkh1. J Cell Sci *129*, 3189-3202.
S17. Mulvihill, D.P., Petersen, J., Ohkura, H., Glover, D.M., and Hagan, I.M. (1999). Plo1 kinase recruitment to the spindle pole body and its role in cell division in Schizosaccharomyces pombe. Mol Biol Cell *10*, 2771-2785.
S18. Asakawa, H., Yang, H.J., Yamamoto, T.G., Ohtsuki, C., Chikashige, Y., Sakata-Sogawa, K., Tokunaga, M., Iwamoto, M., Hiraoka, Y., and Haraguchi, T. (2014). Characterization of nuclear pore complex components in fission yeast Schizosaccharomyces pombe. Nucleus *5*, 149-162.
S19. Grandin, N., and Charbonneau, M. (2002). Mac1, a fission yeast transmembrane protein localizing to the poles and septum, is required for correct cell separation at high temperatures. Biol Cell *94*, 127-137.
S20. Villar-Tajadura, M.A., Coll, P.M., Madrid, M., Cansado, J., Santos, B., and Perez, P. (2008). Rga2 is a Rho2 GAP that regulates morphogenesis and cell integrity in S. pombe. Mol Microbiol *70*, 867-881.
S21. McDonald, N.A., Lind, A.L., Smith, S.E., Li, R., and Gould, K.L. (2017). Nanoscale architecture of the Schizosaccharomyces pombe contractile ring. Elife *6*.
S22. Papadopoulou, K., Ng, S.S., Ohkura, H., Geymonat, M., Sedgwick, S.G., and McInerny, C.J. (2008). Regulation of gene expression during M-G1-phase in fission yeast through Plo1p and forkhead transcription factors. J Cell Sci *121*, 38-47.
S23. Zaaijer, S., Shaikh, N., Nageshan, R.K., and Cooper, J.P. (2016). Rif1 Regulates the Fate of DNA Entanglements during Mitosis. Cell Rep *16*, 148-160.
S24. Deng, L., Kabeche, R., Wang, N., Wu, J.Q., and Moseley, J.B. (2014). Megadalton-node assembly by binding of Skb1 to the membrane anchor Slf1. Mol Biol Cell *25*, 2660-2668.
S25. Trautmann, S., Wolfe, B.A., Jorgensen, P., Tyers, M., Gould, K.L., and McCollum, D. (2001). Fission yeast Clp1p phosphatase regulates G2/M transition and coordination of cytokinesis with cell cycle progression. Curr Biol *11*, 931-940.
S26. Nakaseko, Y., Goshima, G., Morishita, J., and Yanagida, M. (2001). M phase-specific kinetochore proteins in fission yeast: microtubule-associating Dis1 and Mtc1 display rapid separation and segregation during anaphase. Curr Biol *11*, 537-549.
S27. Singh, N.S., Shao, N., McLean, J.R., Sevugan, M., Ren, L., Chew, T.G., Bimbo, A., Sharma, R., Tang, X., Gould, K.L., et al. (2011). SIN-inhibitory phosphatase complex promotes Cdc11p dephosphorylation and propagates SIN asymmetry in fission yeast. Curr Biol *21*, 1968-1978.
S28. Tanabe, K., Ito, N., Wakuri, T., Ozoe, F., Umeda, M., Katayama, S., Tanaka, K., Matsuda, H., and Kawamukai, M. (2003). Sla1, a Schizosaccharomyces pombe homolog of the human La protein, induces ectopic meiosis when its C terminus is truncated. Eukaryot Cell *2*, 1274-1287.
S29. Limbo, O., Chahwan, C., Yamada, Y., de Bruin, R.A., Wittenberg, C., and Russell, P. (2007). Ctp1 is a cell-cycle-regulated protein that functions with Mre11 complex to control double-strand break repair by homologous recombination. Mol Cell *28*, 134-146.
S30. Vashisht, A.A., Kennedy, P.J., and Russell, P. (2009). Centaurin-like protein Cnt5 contributes to arsenic and cadmium resistance in fission yeast. FEMS Yeast Res *9*, 257-269.

S31. Asp, E., and Sunnerhagen, P. (2003). Mkp1 and Mkp2, two MAPKAP-kinase homologues in Schizosaccharomyces pombe, interact with the MAP kinase Sty1. Mol Genet Genomics *268*, 585-597.
S32. Leverson, J.D., Huang, H.K., Forsburg, S.L., and Hunter, T. (2002). The Schizosaccharomyces pombe aurora-related kinase Ark1 interacts with the inner centromere protein Pic1 and mediates chromosome segregation and cytokinesis. Mol Biol Cell *13*, 1132-1143.
S33. Takeda, K., Yoshida, T., Kikuchi, S., Nagao, K., Kokubu, A., Pluskal, T., Villar-Briones, A., Nakamura, T., and Yanagida, M. (2010). Synergistic roles of the proteasome and autophagy for mitochondrial maintenance and chronological lifespan in fission yeast. Proc Natl Acad Sci U S A *107*, 3540-3545.
S34. Gaits, F., Degols, G., Shiozaki, K., and Russell, P. (1998). Phosphorylation and association with the transcription factor Atf1 regulate localization of Spc1/Sty1 stress-activated kinase in fission yeast. Genes Dev *12*, 1464-1473.
S35. Cadou, A., Couturier, A., Le Goff, C., Soto, T., Miklos, I., Sipiczki, M., Xie, L., Paulson, J.R., Cansado, J., and Le Goff, X. (2010). Kin1 is a plasma membrane-associated kinase that regulates the cell surface in fission yeast. Mol Microbiol *77*, 1186-1202.
S36. Hagan, I., and Yanagida, M. (1995). The product of the spindle formation gene sad1+ associates with the fission yeast spindle pole body and is essential for viability. J Cell Biol *129*, 1033-1047.
S37. Furuya, K., Takahashi, K., and Yanagida, M. (1998). Faithful anaphase is ensured by Mis4, a sister chromatid cohesion molecule required in S phase and not destroyed in G1 phase. Genes Dev *12*, 3408-3418.
S38. King, M.C., Drivas, T.G., and Blobel, G. (2008). A network of nuclear envelope membrane proteins linking centromeres to microtubules. Cell *134*, 427-438.
S39. Valbuena, N., and Moreno, S. (2010). TOR and PKA pathways synergize at the level of the Ste11 transcription factor to prevent mating and meiosis in fission yeast. PLoS One *5*, e11514.
S40. Tay, Y.D., Leda, M., Goryachev, A.B., and Sawin, K.E. (2018). Local and global Cdc42 guanine nucleotide exchange factors for fission yeast cell polarity are coordinated by microtubules and the Tea1-Tea4-Pom1 axis. J Cell Sci *131*.
S41. Rajagopalan, S., and Balasubramanian, M.K. (2002). Schizosaccharomyces pombe Bir1p, a nuclear protein that localizes to kinetochores and the spindle midzone, is essential for chromosome condensation and spindle elongation during mitosis. Genetics *160*, 445-456.
S42. Birot, A., Eguienta, K., Vazquez, S., Claverol, S., Bonneu, M., Ekwall, K., Javerzat, J.P., and Vaur, S. (2017). A second Wpl1 anti-cohesion pathway requires dephosphorylation of fission yeast kleisin Rad21 by PP4. EMBO J *36*, 1364-1378.
S43. Kinugasa, Y., Hirano, Y., Sawai, M., Ohno, Y., Shindo, T., Asakawa, H., Chikashige, Y., Shibata, S., Kihara, A., Haraguchi, T., et al. (2019). The very-long-chain fatty acid elongase Elo2 rescues lethal defects associated with loss of the nuclear barrier function in fission yeast cells. J Cell Sci *132*.
S44. Gregan, J., Lindner, K., Brimage, L., Franklin, R., Namdar, M., Hart, E.A., Aves, S.J., and Kearsey, S.E. (2003). Fission yeast Cdc23/Mcm10 functions after pre-replicative complex formation to promote Cdc45 chromatin binding. Mol Biol Cell *14*, 3876-3887.
S45. Taylor, M., Moore, K., Murray, J., Aves, S.J., and Price, C. (2011). Mcm10 interacts with Rad4/Cut5(TopBP1) and its association with origins of DNA replication is dependent on Rad4/Cut5(TopBP1). DNA Repair (Amst) *10*, 1154-1163.

S46. Jin, Y., Rodriguez, A.M., Stanton, J.D., Kitazono, A.A., and Wyrick, J.J. (2007). Simultaneous mutation of methylated lysine residues in histone H3 causes enhanced gene silencing, cell cycle defects, and cell lethality in Saccharomyces cerevisiae. Mol Cell Biol *27*, 6832-6841.

S47. Albert, B., Colleran, C., Leger-Silvestre, I., Berger, A.B., Dez, C., Normand, C., Perez-Fernandez, J., McStay, B., and Gadal, O. (2013). Structure-function analysis of Hmo1 unveils an ancestral organization of HMG-Box factors involved in ribosomal DNA transcription from yeast to human. Nucleic Acids Res *41*, 10135-10149.

S48. Kume, K., Kubota, S., Koyano, T., Kanai, M., Mizunuma, M., Toda, T., and Hirata, D. (2013). Fission yeast leucine-rich repeat protein Lrp1 is essential for cell morphogenesis as a component of the morphogenesis Orb6 network (MOR). Biosci Biotechnol Biochem *77*, 1086-1091.

S49. Yamashita, A., Takayama, T., Iwata, R., and Yamamoto, M. (2013). A novel factor Iss10 regulates Mmi1-mediated selective elimination of meiotic transcripts. Nucleic Acids Res *41*, 9680-9687.

S50. Kimura, M., Suzuki, H., and Ishihama, A. (2002). Formation of a carboxy-terminal domain phosphatase (Fcp1)/TFIIF/RNA polymerase II (pol II) complex in Schizosaccharomyces pombe involves direct interaction between Fcp1 and the Rpb4 subunit of pol II. Mol Cell Biol *22*, 1577-1588.

S51. Huang, Y., McGillicuddy, E., Weindel, M., Dong, S., and Maraia, R.J. (2003). The fission yeast TFIIB-related factor limits RNA polymerase III to a TATA-dependent pathway of TBP recruitment. Nucleic Acids Res *31*, 2108-2116.

S52. Hou, H., Zhou, Z., Wang, Y., Wang, J., Kallgren, S.P., Kurchuk, T., Miller, E.A., Chang, F., and Jia, S. (2012). Csi1 links centromeres to the nuclear envelope for centromere clustering. J Cell Biol *199*, 735-744.

S53. Knezevic, I., Gonzalez-Medina, A., Gaspa, L., Hidalgo, E., and Ayte, J. (2018). The INO80 complex activates the transcription of S-phase genes in a cell cycle-regulated manner. FEBS J *285*, 3870-3881.

S54. Aoi, Y., Kawashima, S.A., Simanis, V., Yamamoto, M., and Sato, M. (2014). Optimization of the analogue-sensitive Cdc2/Cdk1 mutant by in vivo selection eliminates physiological limitations to its use in cell cycle analysis. Open Biol *4*.

S55. Kamenz, J., Mihaljev, T., Kubis, A., Legewie, S., and Hauf, S. (2015). Robust Ordering of Anaphase Events by Adaptive Thresholds and Competing Degradation Pathways. Mol Cell *60*, 446-459.

S56. Bridge, A.J., Morphew, M., Bartlett, R., and Hagan, I.M. (1998). The fission yeast SPB component Cut12 links bipolar spindle formation to mitotic control. Genes Dev *12*, 927-942.

S57. Grallert, A., Chan, K.Y., Alonso-Nunez, M.L., Madrid, M., Biswas, A., Alvarez-Tabares, I., Connolly, Y., Tanaka, K., Robertson, A., Ortiz, J.M., et al. (2013). Removal of centrosomal PP1 by NIMA kinase unlocks the MPF feedback loop to promote mitotic commitment in S. pombe. Curr Biol *23*, 213-222.

S58. Petersen, J., and Hagan, I.M. (2005). Polo kinase links the stress pathway to cell cycle control and tip growth in fission yeast. Nature *435*, 507-512.
